# Prevalence, Virulence and Antimicrobial Resistance of *Vibrio cholerae* in Aquatic Products and Aquaculture Environment in Shanghai

**DOI:** 10.3390/foods14223824

**Published:** 2025-11-07

**Authors:** Yingqi Li, Junjun Liu, Xin Yang, Weiqing Lan, Yong Zhao, Xiaohong Sun

**Affiliations:** 1 College of Food Science and Technology, Shanghai Ocean University, Shanghai 201306, China; 13167063852@163.com (Y.L.);; 2 Shanghai Aquatic Products Processing and Storage Engineering Technology Research Center, Shanghai 201306, China; 3 Laboratory of Quality & Safety Risk Assessment for Aquatic Products on Storage and Preservation (Shanghai), Ministry of Agriculture and Rural Affairs, Shanghai 201306, China

**Keywords:** *V. cholerae*, aquaculture, virulence genes, multidrug resistance, non-O1/O139, ERIC-PCR

## Abstract

In this study, we isolated 214 *Vibrio cholerae* strains from aquatic (shrimp, crab, grass carp, and crucian carp) and their cultured environment in Shanghai, China. The virulence, serotype, and antimicrobial susceptibility were tested, and polymerase chain reaction (PCR) was used to detect antimicrobial resistance genes. Enterobacterial repetitive intergenic consensus polymerase chain reaction (ERIC-PCR) was employed for cluster analysis of the isolated strains. The results showed that *V. cholerae* was found in 47.9% (114/238) of aquatic samples, with the highest detection rate in shrimp (81.1%), and the detection rate was highest in summer (70.0%). Most of the strains were non-O1/O139 groups, and virulence genes *rtxC* and *hap* had the highest detection rates of 92.5% and 91.1%. Of the 214 isolates, 69.6% were multidrug-resistant (MDR). The resistance rate of *V. cholerae* to sulfamethoxazole, ampicillin, and erythromycin was 97.2%, 85.5%, and 70.1%, and that to imipenem, tetracyclines, and aminoglycosides was less than 5%. The MAR index ranged from 0.05 to 0.47. When *V. cholerae* was screened for antimicrobial resistance genes, β-lactams *CARB*, chloramphenicol *floR*, and sulfonamides *sul2* were found in 19.6%, 7.9%, and 6.5% of isolates, respectively. The results of ERIC-PCR clustering showed that the isolates had a high degree of genetic diversity. The widespread distribution of virulent and MDR *V. cholerae* strains poses a potential threat to food safety and public health, calling for improved monitoring and control measures in the aquaculture industry.

## 1. Introduction

As a highly adaptable Gram-negative bacterium, *Vibrio cholerae* inhabits aquatic products and marine environments across a wide global range of temperatures and salinities [1]. *V. cholerae* can cause cholera, a severe diarrheal disease that can be quickly fatal if untreated, and it is typically transmitted via contaminated water and person-to-person contact [2]. The increasing number of human infections caused by *V. cholerae* poses a serious threat to humans and the aquaculture industry [3]. Despite improvements in water quality, sanitation, and hygiene, as well as in the clinical treatment of cholera, the disease is still estimated to cause about 100,000 deaths every year [4].

Cholera, caused by the infection of toxigenic *V. cholerae* to humans, is a life-threatening diarrheal disease with epidemic and pandemic potential. *V. cholerae*, both O1 and O139 serogroups, produces a potent enterotoxin (cholera toxin) responsible for the lethal symptoms of the disease [5]. There are many virulence factors of *V. cholerae*. Two main virulence factors, cholera toxin (CT) and Toxin Coregulated Pilus (TCP), have been found in the O1 and O139 serotypes of *V. cholerae* that cause cholera epidemics [6]. CT causes diarrhea in patients, and TCP enables bacteria to adhere to the intestinal mucosa and form microcolonies [7]. Other virulence-related factors such as zonula occludens toxin (*zot*), accessory cholera enterotoxin (ace), repeats in toxin gene cluster (rtxA-D), hemolysin (hlyA), thermolabile hemolysin (tlh), heat-stable enterotoxin (stn/sto), hemagglutinin protease (hapA), outer membrane protein (ompU), mannose-sensitive hemagglutination pili (mshA), putative type IV pilus (pil), and ToxR regulatory proteins, etc., are also involved in the pathogenic process [6,8,9]. Although the mechanisms by which most *V. cholerae* causes diseases in aquatic products may differ from those in humans, it is still believed that there is a possibility of zoonotic diseases caused by toxic *V. cholerae* [8]. It has been confirmed that *V. cholerae* can be transmitted from the environment to humans through water sources and seafood [10].

In recent decades, the emergence of antimicrobial resistance in pathogens has been observed worldwide and has caused public health concerns. Antimicrobial resistance can occur frequently in aquaculture settings due to the widespread and disorderly use of antimicrobials. Antimicrobial-resistant strains could be harmful to human health because mobile genetic elements can spread resistance genes to other human pathogens through the food chain [11]. Antimicrobial resistance genes and pathogenic bacteria are often accompanied by wastewater discharge into aquatic cultural environments. Capkin et al. found that 92.45% of *Escherichia coli* isolated from trout farms was resistant to sulfamethoxazole, followed by ampicillin (47.79%) and imipenem (28.93%). At least one resistance gene was found in 70.8% of the bacteria, and 66.6% of the bacteria had two or more resistance genes. Approximately 36.54% of the bacteria that contained plasmids were able to transfer them to other bacteria [12]. Wu et al. detected bacteria in aquatic products collected from Zhejiang, China, of which 109 strains (80.15%) showed resistance to at least one antimicrobial, and 38 antimicrobial resistance genes (ARGs) were identified across all samples [13]. In addition, a previous study has shown that *V. cholerae* isolated from shrimp farming environments exhibited resistance rates of up to 46.6% for cephazolin, 30.7% for streptomycin, and 11.4% for ampicillin [14]. These findings suggest that aquatic environments may play a significant role in the development of antimicrobial resistance and the transmission of resistance genes among bacteria. Thus, aquatic bacteria, including pathogenic *V. cholerae*, can act as a repository of resistance genes and may be important to the transmission and evolution of antimicrobial genes in the aquatic cultured environment [15]. Antimicrobial resistance persists in aquatic systems, and its residues and cross-domain transmission pose a dual threat to ecosystems and human health. Aquatic foods may serve as carriers, guiding antimicrobial-resistant symbionts into the human gastrointestinal system, where they interact with the gut microbiota and spread antimicrobial resistance. The emergence of multiple-antimicrobial-resistant strains raises significant concerns regarding seafood safety and human health [16].

To support the consumption of aquatic products and provide guidance on the scientific use of medicines, it is crucial to improve the understanding of the occurrence and antimicrobial susceptibility patterns of *V. cholerae* originating from aquatic sources. Therefore, this study aimed to evaluate the occurrence and antimicrobial susceptibility patterns of *V. cholerae* strains in shrimp, grass carp, crucian carp, crab, and cultured environment samples from different districts in Shanghai. In addition, the virulence genes, antimicrobial genes, and genetic diversity of *V. cholerae* isolates were determined. These findings could help to predict potential human health risks associated with *V. cholerae* exposure and provide basic data for subsequent studies of resistance mechanisms.

## 2. Materials and Methods

### 2.1. Sample Collection

From June 2020 to October 2021, 238 aquatic product and aquaculture environment samples (cultured water and sediment of the corresponding aquatic products) were collected monthly from 27 aquaculture farms in 8 districts of Shanghai. Four categories of aquatic products, including shrimp, grass carp, crucian carp, and crab, were collected from farms. The types of shrimp culture were further categorized into greenhouse and open-air culture. The samples were pretreated as follows: For shrimp, the body surface was rinsed with tap water, and the excess water was removed. Sterile scissors were used to remove the shrimp shell, and the shrimp pancreas was picked out and put in a sterile bag (Interscience, Saint Nom la Bretèche, France). Grass carp and crucian carp were similarly rinsed; after drying, the fish’s belly was cut open with a sterile knife, and intestinal and gill contents were transferred to a sterile bag. Crabs were rinsed and dried, and then the shell was removed, and visceral and gill contents were collected in a sterile bag. After pretreatment of the collected samples, 25 mL (g) each of water/mud/fishery samples was placed in a homogenizing bag containing 225 mL of Alkaline Peptone Water (APW; Beijing Land Bridge Technology Co., Ltd., Beijing, China) and beat for 2 min using a BagMixer 400 CC (Interscience, Saint Nom la Bretèche, France) to form sample homogenization for later use. A total of 82 aquaculture samples were collected from each farm, including 37 shrimp, 35 fishes, and 10 crabs; 79 cultured water samples and 77 cultured sediment samples were collected at the same time. The collected samples were categorized according to sample types, quarters, and districts (Table A1). In addition, water-quality-related information (salinity, temperature, pH) was measured by a DSM-5 salinity meter (Lichen Scientific Instruments Co., Ltd., Shanghai, China), 2-3799-01 electronic thermometer (AS ONE, Shanghai, China), and PH-100pro pH meter (Lichen Scientific Instruments Co., Shanghai, China) for some of the farms [17].

### 2.2. Analysis of Vibrio Species

The strain isolation method referred to the “Testing methods for *Vibrio cholerae* in imported and exported food” (SN/T 1002-2010 [18]). For detection of *Vibrio* species, homogenized samples were transferred to 500 mL conical flasks and incubated overnight at 37 °C (8–18 h). The bacteria were picked with an inoculating loop and streaked on thiosulfate citrate bile salts sucrose agar culture medium (TCBS; Beijing Land Bridge Technology Co., Ltd., Beijing, China) plates for 16–24 h. Subsequently, 3–5 suspected colonies of *V. cholerae* were picked from each plate and streaked on TCBS plates for 16–24 h. One suspected colony was picked from each plate and preserved in 25% glycerol (Sigma-Aldrich, Shanghai, China) at minus 80 degrees in a refrigerator pending further verification.

Genomic DNA was extracted from bacterial colonies using a Wizard Genomic DNA extraction kit (Promega, Madison, WI, USA) according to the manufacturer’s instruction. Referring to Fu et al., the specific gene we selected for PCR was *lolB* (lolB-F: TGG GAG CAG CGT CCA TTG TG, lolB-R: CAA TCA CAC CAA GTC ACT C, 516 bp), with *V. cholerae* GIM 1.449 (*lolB*^+^) and *V. cholerae* CICC 23794 (*lolB*^+^) as positive control strains [19]. In addition, the serotype (O1 and O139) gene primers, product sizes, and annealing temperatures are shown in Table A2. The 25 μL PCR reaction system consisted of 12.5 μL of mix, 9.5 μL of dd water, 10 μmol/L of upstream and 1 μL of downstream primers each, and 1 μL of template. PCR amplification products were determined by 1.0% agarose gel electrophoresis.

### 2.3. Identification of Virulence Genes and Antimicrobial Resistance Genes

The presence of virulence and antimicrobial resistance genes in the *V. cholerae* isolates was determined through TProfessional Standard Thermocyler (Biometra, Göttingen, Germany) with a thermal cycler. The primers and PCR amplification conditions are shown in Table A2. Amplified samples were analyzed by electrophoresis in 1% agarose gel. The gel imaging system was applied for analysis of images.

### 2.4. Antimicrobial Susceptibility Tests of V. cholerae Isolates

The susceptibility of *V. cholerae* to various antimicrobial agents was determined through the micro broth dilution method according to the guidelines of the Clinical and Laboratory Standards Institute (CLSI M45-ED3). The antimicrobial concentrations and antimicrobial susceptibility judgement criteria selected for this study are shown in Table A3. The 17 antimicrobials we use are classified into 9 classes, namely, penicillins, carbapenems, cephalosporins, tetracyclines, chloramphenicol, sulfonamides, macrolides, aminoglycoside, and nitrofurans (Table A3). *Escherichia coli* ATCC 25922 was used as a quality control strain in antimicrobial susceptibility determination. The results were interpreted according to the CLSI M45 2016 edition guidelines for drug sensitivity and recorded as susceptible (S), intermediate (I), and resistant (R). Multiple-antimicrobial resistance (MAR) is defined as a measure indicating the level of antimicrobial resistance in organisms, specifically where an index greater than 0.2 points to origins from high-risk contamination sources. The MAR index of the isolates was defined as x/y, where x represents the number of antimicrobial agents to which the isolate was resistant, and y represents the total number of antimicrobial agents against which an individual isolate was tested [20]. Multidrug-resistant (MDR) was defined as acquired non-susceptibility to at least one agent in three or more antimicrobial categories, extensively drug-resistant (XDR) was defined as non-susceptibility to at least one agent in all but two or fewer antimicrobial categories (i.e., bacterial isolates remain susceptible to only one or two categories), and pandrug-resistant (PDR) was defined as non-susceptibility to all agents in all antimicrobial categories. The antimicrobial resistance pattern abundance (ARPA) was expressed as the total number of resistance spectrum types divided by the total number of strains.

### 2.5. Analysis of the Genetic Diversity of V. cholerae

Enterobacterial repetitive intergenic consensus polymerase chain reaction (ERIC-PCR) primer sequences and reaction conditions were carried out according to Mejdi et al. [21]; ERIC-PCR amplification primers were as follows: Eric1: 5′- ATG TAA GCT CCT GGG GAT TCA C-3′; Eric1: 5′- AAG TAA GTG ACT GGG GTG AGC G-3′; the 25 μL reaction system was as follows: mix 12.5 μL, dd H_2_O 8.5 μL, 10 μmol/L upstream and downstream primers 1 μL each, andtemplate 2 μL. Cycling parameters were as follows: pre-denaturation at 95 °C for 5 min, then cycling 94 °C for 45 s, 52 °C for 1 min, and 72 °C for 1 min for 35 cycles. The final extension was 72 °C for 10 min. The amplification products were electrophoresed by 1% agarose gel (Tsingke, Beijing, China) at 100 V for 45 min, and then the electropherograms were analyzed by the gel imaging system. The presence of bands was recorded as 1, and the absence of bands was recorded as 0. The output results were analyzed using the unweighted pair-group method with arithmetic means (UPGMA) in the NTsys-pc software Version 2.10e (New York, NY, USA).

The Simpson Index is used to express species diversity and is calculated using the following formula:

(1) $$1-\frac{1}{N\left(N\;-\;1\right)}\sum_{j=1}^{S}x_{j}(x_{j}\;-\;1)$$

where N is the total number of strains, S is the total number of ERIC genotypes, and Xj is the number of genes in the jth ERIC genotype.

### 2.6. Data Processing and Analysis

In this paper, data results statistics were processed and produced using the GraphPad Prism software 10.1.2 (San Diego, CA, USA), gel electrophoresis plots were generated using the Geldoc XR+ Gel Imaging system (Hercules, CA, USA), and significant difference calculations were produced using the SPSS 25.0 software (New York, NY, USA). The normal distribution of the data was tested using SPSS, and the significance of the data differences was compared by a *t*-test and analysis of variance (ANOVA). *p* < 0.05 was considered statistically different.

## 3. Results

### 3.1. Water Temperature, pH, and Salinity

**Figure 1:**
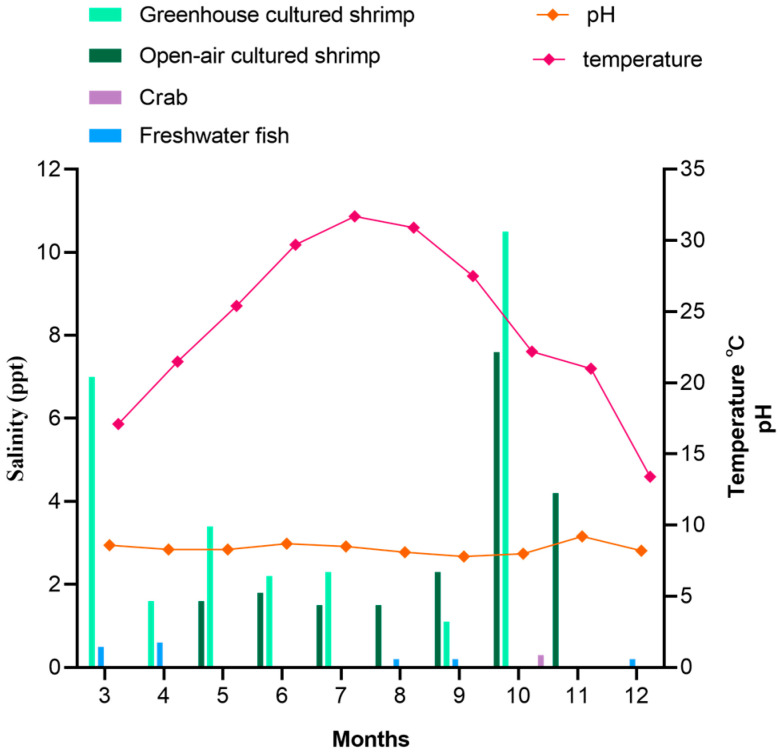
Average physical and chemical information change in farms in different months.

Figure 1 shows the monthly variations in the water temperature, pH, and cultured salinity at different cultivation farms from 2019 to 2020. The monthly mean values of water temperature, pH, and salinity ranged from 13.7 °C to 32.2 °C, 8 to 9, and from 1.1 ppt to 10.5 ppt. The water temperatures were markedly higher during the summer, especially in July. However, the pH did not exhibit large seasonal variations. Moreover, the salinity of shrimp culture environments was significantly higher than that of other aquatic product cultured environments, which was below 1.0 ppt.

### 3.2. Distribution and Serotypes of V. cholerae

Table 1 shows that 214 *V. cholerae* strains were detected in 114 (47.9%) out of 238 aquatic samples. Among them, 159 and 55 *V. cholerae* strains were detected in 81 (71.7%) shrimp samples and 33 (37.9%) freshwater fish samples. *V. cholerae* was not detected in crab samples and its cultured environment. Of the three tested types of aquatic samples, the detection rate was highest in shrimp (81.1%), followed by grass carp (38.9%), and crucian carp (29.4%). There was no significant difference between the *V. cholerae* detection rate in aquatic samples and environmental samples (*p* > 0.05).

The detection rate of *V. cholerae* in different seasons is shown in Table 2. The highest detection rate was 70.0% in summer, followed by spring (54.8%), autumn (39.1%), and winter (12.1%). Among aquatic product samples, the detection rate in greenhouse-cultured shrimp was 100.0% in summer and autumn, with a 75.0% detection rate in spring; the detection rate in open-air-cultured shrimp decreased from 100.0% in spring to 66.7% in autumn. Likewise, the detection rate in grass carp was highest in summer (80.0%). Moreover, in crucian carp, the detection rates were all below 50.0%. Among the environmental samples, the detection rate in the greenhouse-cultured shrimp environmental samples was highest in summer and autumn, while in the open-air-cultured shrimp environmental samples, it was highest in spring. The detection rate of *V. cholerae* in freshwater fish environmental samples was higher than 50.0% in summer and 100.0% in autumn.

Except for three *V. cholerae* strains (*Vc*381, *Vc*382, *Vc*383), which were confirmed as the O139 serotype, the remaining 211 *V. cholerae* strains in the present study were confirmed as non-O1 and non-O139 species. O139-serotype *V. cholerae* strains were isolated from the shrimp aquaculture water samples in summer.

### 3.3. Virulence Genes of V. cholerae Isolates

**Figure 2:**
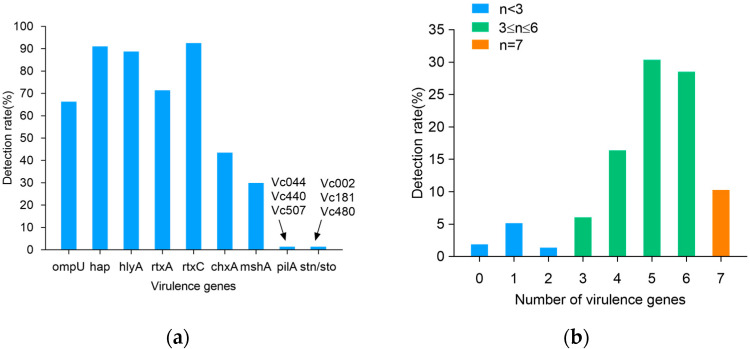
Virulence genes detection rates of 214 *V. cholerae* strains. (**a**) Detection rate of virulence genes; (**b**) number of virulence genes.

Figure 2 summarizes the presence of virulence genes in the *V. cholerae* isolates. A total of nine virulence genes were detected, and the virulence genes *ctxA*, *ctxB*, *ctxAB*, *tcpA*, *ace*, and *zot* were detected in none of the isolates. Among the nine detected genes, *rtxC*, and *hap* had the highest detection rates of 92.5% and 91.1%, followed by *hlyA*, *rtxA*, *ompU*, *chxA*, and *mshA*, with 88.8%, 71.5%, 66.4%, 43.5%, and 29.9% (Figure 2a). The detection rate of *pilA* and *stn/sto* was extremely low, and they were, respectively, detected only in three isolates (*Vc*002, *Vc*181, and *Vc*480, and *Vc*044, *Vc*440, and *Vc*507). As shown in Figure 2b, 214 strains of *V. cholerae* carried several virulence genes, ranging from 0–7. Among them, 8.4% (*n* = 18) of the strains carried less than three virulence genes, the majority of the *V. cholerae* (81.3%) carried four to six virulence genes, and 10.3% (*n* = 22) carried seven virulence genes.

The virulence genes of 210 *V. cholerae* strains exhibited 31 virulence patterns (Table 3). Among them, *V. cholerae*, carrying five virulence genes with a virulence pattern of *hap*, *hlyA*, *rtxA*, *rtxC*, and *ompU*, had the highest percentage of 19.2%. This was followed by 13.1% of *V. cholerae* carrying six virulence genes, with a virulence pattern of *hap*, *hlyA*, *rtxA*, *rtxC*, *ompU*, and *chxA*. Finally, the virulence profiles of the 20 *V. cholerae* strains carrying seven virulence genes were *hap*, *hlyA*, *rtxA*, *rtxC*, *ompU*, *chxA*, and *mshA* in 9.3%. Overall, there was diversity in the pattern of *V. cholerae* virulence gene carriage.

### 3.4. Antimicrobial Resistance Patterns of V. cholerae Isolates and Analysis of V. cholerae Resistance in Different Aquatic Samples

**Figure 3:**
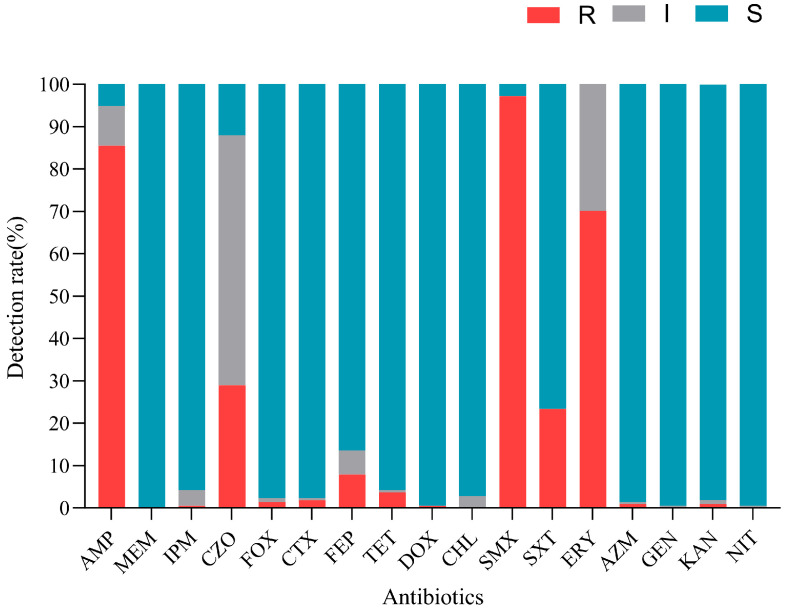
Antimicrobial sensitivity of 214 *V. cholerae* strains.

To determine the antimicrobial resistance patterns of *V. cholerae* isolates, 17 antimicrobials were chosen for antimicrobial susceptibility testing. The results showed that strains were resistant to 13 antimicrobials of seven classes. As shown in Figure 3, among the 214 *V. cholerae* isolates tested, 97.2% were resistant to sulfamethoxazole (SMX), representing the highest resistance to antimicrobials used in this study. In addition, the isolates exhibited relatively high resistance to ampicillin (AMP) (85.5%), erythromycin (ERY) (70.1%), cefazolin (CZO) (29.0%), and cotrimoxazole (SXT) (23.4%). Moreover, among the cephalosporin antimicrobials, the isolates exhibited the highest resistance to CZO (29.0%), followed by cefepime (FEP) (7.9%), and less than 5% of the isolates were resistant to cefoxitin (FOX) and cefotaxime (CTX). Meanwhile, isolates showed low resistance to five antimicrobials (imipenem (IPM), tetracycline (TET), doxycycline (DOX), azithromycin (AZM), and kanamycin (KAN)) (<5%). All isolates were sensitive to meropenem (MEM). A small number of isolates exhibited intermediate resistance to chloromycetin (CHL), gentamycin (GEN), and nitrofurantoin (NIT), and more than 90.0% of isolates were susceptible to IPM, FOX, CTX, AZM, CHL, TET, DOX, GEN, and KAN. The results showed that *V. cholerae* isolated from aquatic products and the culture environment was only resistant to some antimicrobials, and it did not show an all-around high level of antimicrobial resistance.

The strains exhibited 39 resistance patterns, with an antimicrobial resistance pattern abundance (ARPA) of 0.18 and 69.6% of MDR strains (Table 4). In addition, none of the strains were XDR or PDR. The main resistance pattern was AMP-SMX-ERY (*n* = 66), followed by AMP-SMX (*n* = 28) and AMP-CZO-SMX-ERY (*n* = 23).

### 3.5. Antimicrobial Resistance Analysis of V. cholerae in Shrimp and Fish

**Figure 4:**
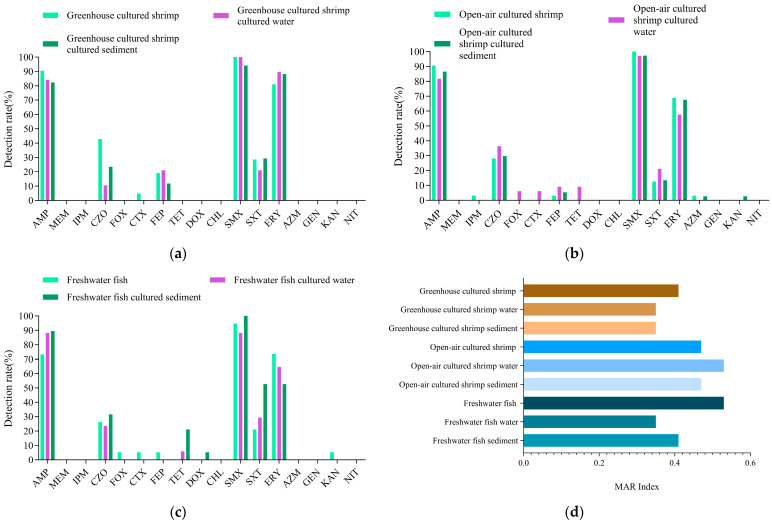
Antimicrobial resistance analysis of *V. cholerae* from shrimp, freshwater fish, and their environmental samples. (**a**) The antimicrobial resistance rate of *V.cholerae* from greenhouse cultured shrimp; (**b**) The antimicrobial resistance rate of *V.cholerae* from Open-air cultured shrimp; (**c**) The antimicrobial resistance rate of *V.cholerae* from freshwater fish; (**d**) The MAR Indexes of *V.cholerae* from shrimp, freshwater fish, and their environmental samples.

To determine *V. cholerae* resistance in shrimp, antimicrobial susceptibility tests for *V. cholerae* were conducted. As shown in Figure 4a, the 57 strains of *V. cholerae* in greenhouse-cultured shrimp, water, and sediment showed varying resistance rates to seven antimicrobials of four classes. Notably, they showed over 80% resistance to AMP, ERY, and SMX, while resistance to CZO, FEP, and SXT ranged from 10% to 50%. Only one strain (*Vc*001) was resistant to CTX. The MAR index was 0.41 for shrimp, 0.35 for water, and 0.35 for sediment, thus suggesting slightly higher contamination in shrimp compared to its cultured environment (Figure 4d).

Figure 4b shows the sensitivity of 102 *V. cholerae* strains from open-air-cultured shrimp, water, and sediment to 12 antimicrobials of seven classes. *V. cholerae* from shrimp was resistant to eight antimicrobials of five classes, while *V. cholerae* from water and sediment were resistant to nine and eight antimicrobials of five classes. *V. cholerae* from shrimp and the environment showed more than 95% resistance to SMX, 50–90% to AMP and ERY, and less than 50% to CZO, FEP, and SXT. *V. cholerae* from shrimp had a 3.1% resistance rate to IPM, FEP, and AZM. *V. cholerae* from water showed resistance to all four generations of cephalosporins, with the highest resistance rate (36.4%) to CZO. Resistance to FOX, CTX, and FEP was 6.1% (*n* = 2), 6.1% (*n* = 2), and 9.1% (*n* = 3). In addition, they also showed 9.1% resistance to TET. *V. cholerae* from sediment had a 2.7% (*n* = 1) resistance rate to AZM and KAN. The MAR indexes of open-air-cultured shrimp, water, and sediment were 0.47, 0.53, and 0.47, indicating higher antimicrobial contamination in water than in shrimp and sediment (Figure 4d).

Figure 4c shows that strains from freshwater fish, water, and sediment were resistant to 11 antimicrobials of six classes, with *V. cholerae* from freshwater fish resistant to 9 antimicrobials of five classes, and *V. cholerae* from the environment resistant to 7 antimicrobials. All strains showed high resistance to AMP, SMX, and ERY (50–100%) and low resistance to CZO and SXT. In addition, they showed resistance to all four generations of cephalosporins, with the highest resistance to first-generation CZO (26.3%) and lower resistance to other generations (5.3%). Resistance to aminoglycoside KAN was 5.3%. Tetracycline-resistant strains were detected only in cultured environmental samples. The MAR index for *V. cholerae* from freshwater fish was 0.53, higher than 0.35 for water and 0.41 for sediment (Figure 4d).

### 3.6. Detection of V. cholerae Drug Resistance Genes

**Figure 5:**
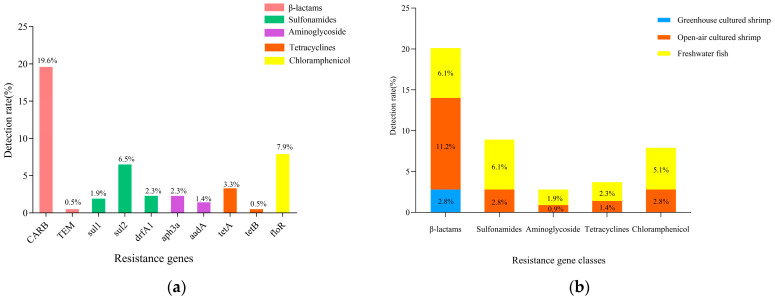
Results of resistance genes of *V. cholerae* in aquatic products and environmental samples. (**a**) Resistance genes; (**b**) resistance gene classes.

A total of 10 drug resistance genes of five classes were detected in 214 *V. cholerae* strains (Figure 5a). β-lactams *CARB*, chloramphenicol *floR*, and sulfonamides *sul2* were detected at 19.6%, 7.9%, and 6.5%. β-lactams (*TEM*), sulfonamides (*sul1*, *drfA1*), aminoglycosides (*aph3a*, *aadA*), and tetracyclines (*tetA*, *tetB*) had resistance gene detection rates below 5%.

As shown in Figure 5b, only β-lactam resistance genes were detected in *V. cholerae* in greenhouse-cultured shrimp. In contrast, five classes of resistance genes were detected in *V. cholerae* in open-air-cultured shrimp and freshwater fish. Among them, β-lactams dominated the resistance genes of *V. cholerae* in open-air-cultured shrimp, with detection rates of 11.2%. In freshwater fish, the detection rate of β-lactam resistance genes and sulfonamide resistance genes were the highest (6.1%), followed by chloramphenicol (5.1%) and tetracyclines (2.3%).

### 3.7. Analysis of Genetic Diversity

**Figure 6:**
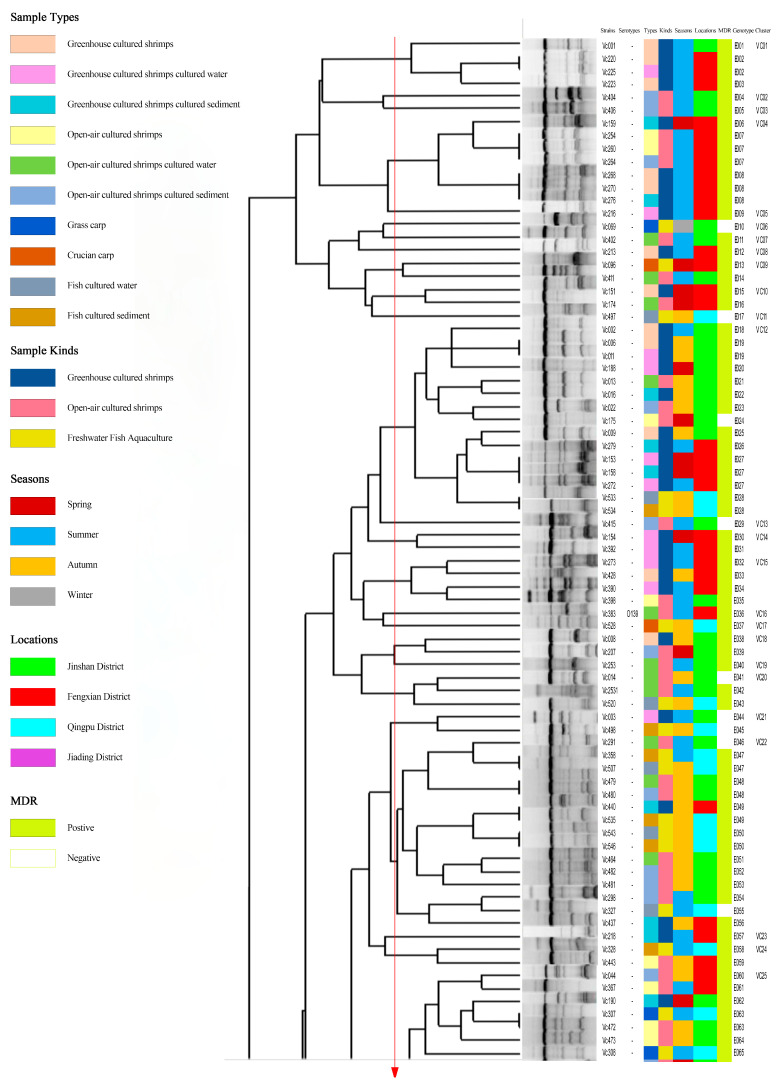

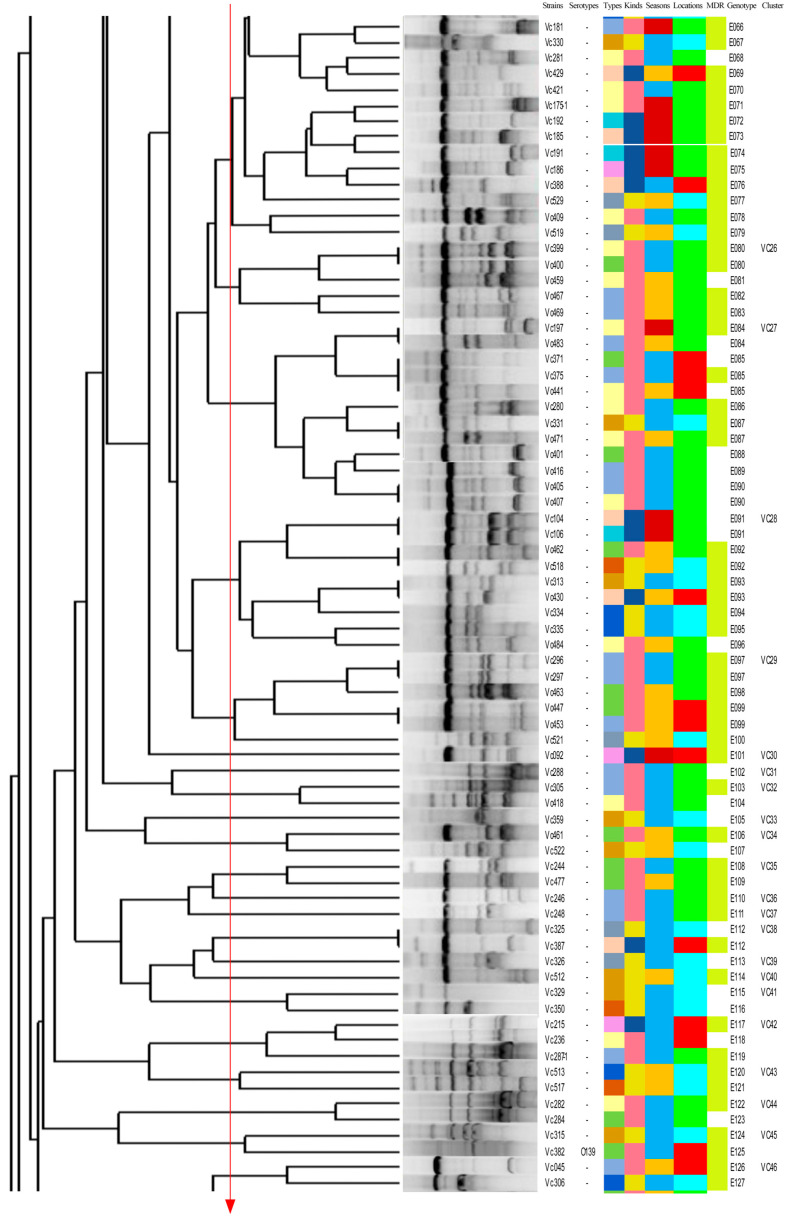

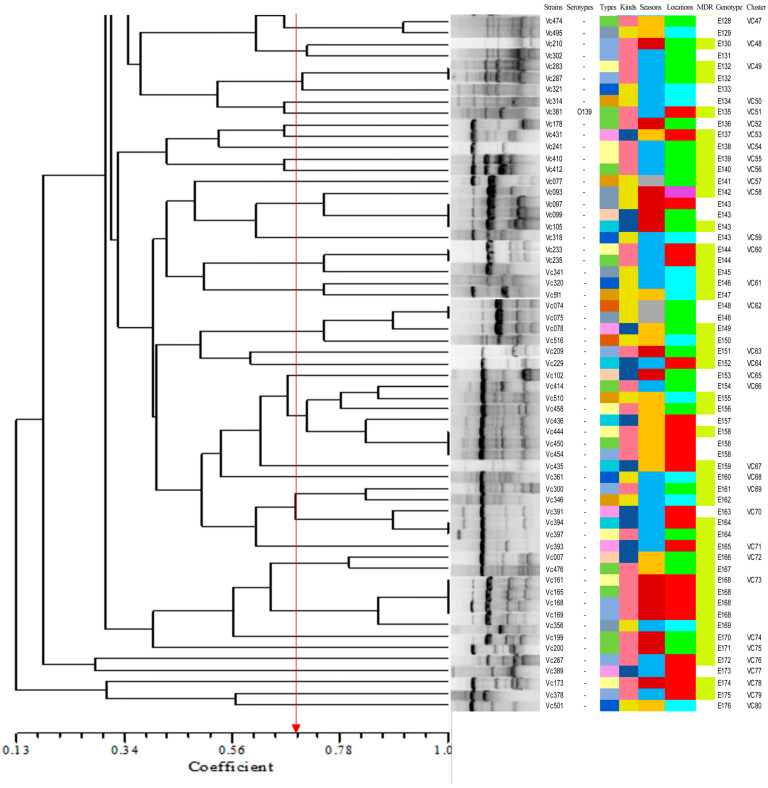
Dendrogram of 214 *V. cholerae* by using ERIC-PCR.

Figure 6 shows the ERIC-PCR clustering results for 214 *V. cholerae* strains, with strain similarity ranging from 13% to 100%, clustering into 177 ERIC genotypes (E001-E177), where 69.2% (148/214) were single genotypes. A total of 26.5% (39/148), 46.9% (69/148), and 27.0% (40/148) of the single genotypes were from greenhouse-cultured shrimp, open-air-cultured shrimp, and freshwater fish; 16.3% (24/148), 50.7% (75/148), 32.0% (47/148), and 1.4% (2/148) of the single genotypes were from spring, summer, and fall. Strains with similarity coefficients ≥0.69 were defined as identical groups (marked by red solid lines). The 177 ERIC genotypes were clustered into 80 distinct groups (*Vc*01-*Vc*80), 43 of which contained only one ERIC genotype. All ERIC genotypes contained less than five strains, with no clear dominant ERIC type; of the 80 groups, group *Vc*25 was the most dominant group, containing 9.8% (21/214) of the strains. There were significant differences between ERIC genotypes and sample types, sampling times, and serotypes (*p* < 0.05). This suggested that the 214 *V. cholerae* strains were highly genetically diverse, showing significant differences in genotypic composition across serotypes, species of aquatic product, and time.

## 4. Discussion

*C*holera caused by *V. cholerae* continues to pose a global threat, despite improvements in sanitation and treatment methods, and it remains a significant public health issue. The emergence and spread of antimicrobial resistance in aquatic environments, coupled with the overuse of antimicrobials in aquaculture, further exacerbate the threat to human health. Therefore, it is necessary to assess the prevalence, antimicrobial susceptibility, pathogenic genes, and genetic diversity of *V. cholerae* in aquatic products and aquaculture environments, which will help evaluate potential health risks and provide foundational data for understanding resistance mechanisms.

*V. cholerae* was detected in all seasons, and the detection rate in different seasons was highest in summer and lowest in winter. There were significant differences in *V. cholerae* detection rates between different seasons (*p* < 0.05). Our study showed that environmental temperature can significantly affect *V. cholerae* abundance. These findings are consistent with previous research showing that *V. cholerae* abundance correlates to temperature, and *Vibrio* species display preferences for warm tropical water in summer [22]. In addition, heavy rainfall during the summer months reduces the salinity of water, which directly affects the detection rate of *V. cholerae*.

In this study, the results suggest that shrimp is more likely to be a host for *V. cholerae*. The main reason may be that compared to freshwater fish and crab culture environments, the salinity of shrimp culture environments is higher. It has been reported that the salinity of an environment significantly correlates with *V. cholerae* incidence [23,24]. Another study has shown that a sharp fall in the cultivable count of *V. cholerae* during monsoon is attributed to reduced salinity [25]. The optimal salinity range for *V. cholerae* growth is 15–25 ppt [26]. In this study, the salinity of the shrimp culture environment was between 1.1 and 10.5 ppt (Figure 1), while salinities in freshwater fish and crab culture environments were generally below 1.0 ppt, which is not favorable for *V. cholerae* growth. Ahmed et al. collected 132 shrimp samples from a market, in which the *V. cholerae* detection rate was 1.5% much lower than this study [27]. This evidence is concerning because it shows that *V. cholerae* is highly prevalent in aquaculture.

Cholera enterotoxin is the primary virulence factor of the disease cholera, which is caused by infection with toxigenic *V. cholerae* serotypes O1 or O139. The other serotypes are collectively known as non-O1/O139 *V. cholerae*. In this study, *V. cholerae* strains were mainly non-O1/O139 serotypes, and the main virulence genes *ctxA/B/AB* and *tcpA* were detected in none of the 214 *V. cholerae* strains. Similarly, a study indicated that very few non-O1/O139 *V. cholerae* isolates from clinical sources had the *TCP* and *CTX* gene cassettes [9]. Most previous studies focused on the harm caused by *V. cholerae* strains of serotypes O1 or O139. However, previous research has shown that four resistance-associated genes (*strB*, *dfrA1*, *sulll*, *SXT*) were confirmed to have a higher prevalence in *V. cholerae* non-O1/non-O139 strains [28]. These results demonstrate a potential threat of non-O1/O139 *V. cholerae* in aquatic products. Therefore, there is a need for continuous testing of non-O1/O139-serotype *V. cholerae* prevalence in aquaculture environmental sources.

The carriage of virulence genes and pathogenicity factors is an indicator of strain virulence assessment. In addition to the main *ctxAB* and *tcpA* virulence genes, other virulence genes play important roles in *V. cholerae* pathogenesis [28]. In this study, the detection rates of the *HAP* and *rtxC* were extremely high, exceeding 90%. The detection of rtxC in the current study is similar to some earlier studies [29,30,31]. Among them, *HAP* encodes a hemagglutinin protease that degrades host cell intercellular adhesion proteins and enhances bacterial invasion capacity [32]. In non-O1O139 *V. cholerae* infections, HAP protease directly causes intestinal epithelial cell detachment and inflammatory responses, leading to watery diarrhea, abdominal pain, and bloody stools [8]. *RtxA* belongs to the same RTX toxin gene cluster as *rtxC*, which disrupts host cell membrane integrity through a perforation mechanism and leads to permanent dissociation of the cytoskeleton [33]. In immunocompromised individuals, RTX toxin may promote bacterial entry into the bloodstream, leading to sepsis. Meyer et al. found that all 29 clinical isolates carrying *rtxA* were associated with extraintestinal infections [34]. The actin cross-linking repeats in the toxin gene cluster (rtx) lead to actin depolymerization when cross-linked with Hep-2, and this causes villi effacement, hemorrhagic colitis, and bloody diarrhea, as reported earlier [35,36]. The detection rate of *hlyA* was relatively high at 71.5%. The prevalence of *hlyA* in our study is similar to that of earlier studies from clinical and environmental samples [31,37,38]. *HlyA* is involved in cellular infection through the modulation of hemolysins, and *hlyA* hemolysin promotes bacterial proliferation by lysing red blood cells to release iron ions [39,40]. The results of this study are consistent with the results of the detection of *V. cholerae* virulence genes isolated from common aquatic products from Shanghai supermarkets by Xu et al. and Fu et al. [6,19]. The detection of these virulence genes suggests that *V. cholerae* has multiple pathogenic potentials: initially, it establishes infection foci through adhesion of bacterial hairs, then releases multiple toxins to disrupt the intestinal barrier function, and ultimately leads to severe diarrhea, electrolyte disorders, and systemic toxicity symptoms [3,41,42]. In particular, the synergistic effect of RTX toxins and hemolysin may aggravate intestinal mucosal damage and systemic inflammatory response [43]. It can be seen that there is diversity in the pattern of *V. cholerae* virulence gene carriage in an aquaculture environment. The detection of multiple related virulence genes of *V. cholerae* constitutes the genetic basis for causing symptoms such as mild gastroenteritis or even severe diarrhea. Non-major virulence genes provide the genetic basis for causing mild gastroenteritis and even severe diarrhea, which need to be guarded against.

Antimicrobial resistance assessment under pathogens and its potential ecology risk is a major worldwide concern for medicine research and development [44]. The results showed that the strains of *V. cholerae* were resistant to 13 antimicrobials of seven classes, indicating that *V. cholerae* had developed multidrug resistance in the aquatic culture environment. This is consistent with previous studies, which have found that *V. cholerae* from aquatic products from seafood markets are highly resistant to penicillin (P), AMP, and amoxicillin (AMOX) [37]. Additionally, Wu et al. found the highest resistance rate in erythromycin (36%) among all tested macrolide antimicrobials in O1/O139 *V. cholerae* [45]. Another study recorded high rates of resistance in environmental *V.cholerae* isolates to beta-lactams (penicillin and 15 isolates for ampicillin) and streptomycin [7]. The high resistance rates (>70%) of SMX, AMP, and ERY can be attributed to their overuse in aquaculture and clinical settings. Dua et al. found that non-O1/O139-serotype *V. cholerae* strains from Indian clinical settings have developed widespread resistance to antimicrobials, including AMP, CHL, TET, and SMX, similar to the results of this study [9]. The 23.4% resistance rate of SXT also suggests that it may be overused. Similar to the results in our study, it has been found that the weighted pooled resistance (WPR) rate of non-O1O139 *V. cholerae* from clinical origins to cotrimoxazole is 27% [45]. Compared to Gram-positive bacteria, Gram-negative bacteria are less permeable, and their outer membrane naturally forms a permeability barrier against antimicrobials [46]. It is worth noting that approximately 75% of antimicrobials used in aquaculture enter the environment through feces and feed and accumulate there, potentially driving the development of bacterial resistance [47]. In this study, there were different levels of resistance rates (<5%) to main types of antimicrobials (DOX and CTX), which suggested that there was a certain degree of antimicrobial pollution in the cultured environment. On the whole, the sensitivity of *V. cholerae* to DOX, IPM, MEM, CHL, and other first-line drugs for clinical treatment was higher than 90%. Tetracyclines have been the most effective antimicrobial class in treating cholera for a long time [45]. The sensitivity rate of aminoglycosides (GEN, KAN), macrolides (AZM), nitrofurans (NIT), and second-, third-, and fourth-generation cephalosporins (FOX, CTX, FEP), except CZO, was higher than 85%, which provides multiple options for clinical medication.

The ranking of *V. cholerae* antimicrobial resistance levels in different aquatic products is as follows: greenhouse shrimp > grass carp > open-air shrimp = crucian carp. The MAR index for all strains is higher than 0.2, indicating that *V. cholerae* in the current aquaculture environment is exposed to high levels of antimicrobial contamination. Open-air-cultured shrimp require particular attention. Due to human activities, bacterial resistance is continuously evolving. Therefore, regular monitoring of antimicrobial resistance in aquaculture areas is crucial for ensuring the quality and safety of aquatic products.

This study detected 10 drug resistance genes in five classes. Among these, the detection rate for the β-lactam resistance gene *CARB* was the highest (19.6%), and that for the β-lactam resistance gene *blaTEM* was 0.5%. The resistance gene *CARB* was found to be responsible for penicillin resistance; it is one of the class A β-lactamases that exhibit a broad substrate hydrolysis profile against penicillins [48]. In addition, the resistance rate of penicillin antimicrobial AMP was extremely high in this study. The detection rate of the chloramphenicol resistance gene *floR* was 7.9%, corresponding to the low resistance rate of chloramphenicol CHL (<5%). In addition, the detection rates of sulfonamide resistance genes sul2, drfA1, and sul1 were 6.5%, 2.3%, and 1.9%, respectively, while the resistance rate of sulfonamide antimicrobials was extremely high. This suggests there might be other antimicrobial resistance mechanisms such as efflux pump (either singly or in synergy with resistance genes) to archive antimicrobials resistance. The energy-dependent efflux of multiple antimicrobial agents from bacterial cells of the *Vibrio* spp. is a widely recognized resistance mechanism [49]. In addition, the low detection rate of tetracycline and chloramphenicol resistance genes corresponded to the low resistance rate. In summary, this non-corresponding relationship may be due to the wide variety of drug resistance genes, the existence of multiple alleles, and the fact that *V. cholerae* has a variety of drug resistance mechanisms, resulting in different correlations between drug resistance phenotypes and genotypes. Previous studies have shown that the emergence of *V. cholerae* resistance is mainly promoted by autonomously transmitted, autonomously replicated plasmids or horizontal gene transfer (HGT), which integrate mobile genetic elements [46]. Therefore, it is still necessary to pay attention to the spread of antimicrobial resistance and drug resistance of strains.

Many studies have shown that ERIC-PCR exhibits an excellent typing effect in foodborne pathogens and has been widely used in the study of *V. cholerae* molecular typing [6,19,50]. In our study, 214 *V. cholerae* strains in the aquaculture environment of Shanghai were scattered, and the homology was low. The differences in sample type and isolation time of the strains contained in the same genotype showed that there was a genetic relationship between *V. cholerae* isolates from different aquatic products. Through ERIC-PCR, 214 *V. cholerae* strains were divided into 80 different populations (*Vc*01-*Vc*80), of which 53.8% (43/70) contained only one ERIC genotype. Most of the populations and ERIC genotypes contained no more than five strains, indicating that *V. cholerae* in the aquatic cultural environment has a high genetic diversity.

## 5. Conclusions

*V. cholerae* strains are of particular concern because they pose a major threat to human health worldwide, primarily through the consumption of raw or undercooked seafood. *V. cholerae* was widely distributed across different water samples in Shanghai, with the highest detection rate in shrimp samples and peak prevalence during summer. The vast majority of serotypes were non-O1/O139. *V. cholerae* exhibited high resistance to the penicillin antimicrobial AMP, cephalosporin antimicrobial CZO, sulfonamide antimicrobials SMX and SXT, and macrolide antimicrobial ERY, without indicating high antimicrobial contamination levels. These several antimicrobials already have relatively high pollution levels in the aquaculture environment and are not suitable for continued use. Virulence genes of *rtxC*, *hap*, *hlyA*, and *rtxA* were highly prevalent. *V. cholerae* exhibited high genetic diversity, with significant differences in genotypic composition across serotypes, sample types, and seasons. Our study enriches the epidemic research data of *V. cholerae* in the aquaculture environment and is of certain significance for the continuous monitoring and risk factor identification of *V. cholerae* contamination in the aquaculture environment.

## Figures and Tables

**Table 1:** Distribution of *V. cholerae* in 238 aquatic samples.

Type of Samples	Total Number of Samples	Positive Number of Samples (%)
Greenhouse-cultured shrimp	13	12 (92.3)
Greenhouse-cultured shrimp culture water	13	10 (76.9)
Greenhouse-cultured shrimp culture sediment	13	9 (69.2)
Open-air-cultured shrimp	24	18 (75.0)
Open-air-cultured shrimp culture water	25	17 (68.0)
Open-air-cultured shrimp culture sediment	25	15 (60.0)
Grass carp	18	7 (38.9)
Crucian carp	17	5 (29.4)
Fish culture water	27	11 (40.7)
Fish culture sediment	25	10 (40.0)
Crab	10	0 (0.0)
Crab culture water	14	0 (0.0)
Crab culture sediment	14	0 (0.0)
Total	238	114 (47.9)

**Table 2:** Detection rates of *V. cholerae* in samples from different seasons.

Type of Samples	Detection Rate of *V. cholerae* in Different Quarters (%)			
	Spring	Summer	Autumn	Winter
Greenhouse-cultured shrimp	75.0	100.0	100.0	-
Greenhouse-cultured shrimp culture water	60.0	100.0	66.7	-
Greenhouse-cultured shrimp culture sediment	60.0	80.0	66.7	-
Open-air-cultured shrimp	100.0	71.4	66.7	-
Open-air-cultured shrimp culture water	100.0	64.3	57.1	-
Open-air-cultured shrimp culture sediment	75.0	50.0	71.4	-
Grass carp	0.0	80.0	40.0	20.0
Crucian carp	33.3	33.3	40.0	16.7
Fish culture water	33.3	60.0	100.0	9.1
Fish culture sediment	0.0	80.0	100.0	9.1
Total	54.8	70.0	39.1	12.1

**Table 3:** Virulence patterns of 214 *V. cholerae* strains.

Virulence Pattern	Number of Virulence Genes	Number of Strains	Detection Rate (%)
*rtxA*	1	3	1.4
*rtxC*		1	0.5
*ompU*		7	3.3
*rtxA-ompU*	2	1	0.5
*hlyA-ompU*		1	0.5
*rtxC-ompU*		1	0.5
*hap-rtxC-chxA*	3	1	0.5
*hap-hlyA-rtxC*		12	5.6
*hap-hlyA-rtxC-chxA*	4	8	3.7
*hap-hlyA-rtxA-rtxC*		12	5.6
*hap-hlyA-rtxC-mshA*		1	0.5
*hap-hlyA-rtxC-ompU*		6	2.8
*hap-hlyA-rtxC-mshA*		5	2.3
*hap-hlyA-rtxC-stn/sto*		1	0.5
*hap-rtxA-rtxC-chxA*		2	0.9
*hap-hlyA-rtxC-ompU-stn/sto*	5	1	0.5
*hap-hlyA-rtxA-rtxC-ompU*		41	19.2
*hlyA-rtxA-rtxC-ompU-chxA*		1	0.5
*hap-hlyA-rtxA-rtxC-mshA*		3	1.4
*hap-hlyA-rtxC-ompU-chxA*		5	2.3
*hap-rtxA-rtxC-chxA-mshA*		1	0.5
*hap-hlyA-rtxA-rtxC-chxA*		13	6.1
*hap-hlyA-rtxA-rtxC-chxA-mshA*	6	4	1.9
*hap-hlyA-rtxA-rtxC-ompU-chxA*		28	13.1
*hap-hlyA-rtxA-rtxC-ompU-mshA*		19	8.9
*hap-hlyA-rtxA-rtxC-chxA-pilA*		1	0.5
*hap-rtxA-rtxC-ompU-mshA-pilA*		1	0.5
*hap-hlyA-rtxC-ompU-chxA-mshA*		8	3.7
*hap-hlyA-rtxA-rtxC-ompU-chxA-mshA*	7	20	9.3
*hap-hlyA-rtxA-rtxC-ompU-mshA-pilA*		1	0.5
*hap-hlyA-rtxC-ompU-chxA-mshA-stn/sto*		1	0.5

**Table 4:** Antimicrobial resistance patterns of 214 *V. cholerae* strains.

Resistance Pattern	Number of Antimicrobials	Number of Antimicrobial Classes	Number of Strains	MAR Index
SMX	1	1	7	0.05
AMP		1	1	
CZO-SMX	2	2	1	0.11
AMP-SMX		2	28	
SMX-ERY		2	9	
AMP-ERY		2	3	
SMX-SXT		1	2	
AMP-CZO-SMX	3	3	8	0.16
CZO-SMX-ERY		3	2	
AMP-SMX-SXT		2	6	
CZO-FEP-SMX		2	1	
AMP-CZO-ERY		3	2	
SMX-SXT-ERY		2	7	
AMP-SMX-ERY		3	66	
AMP-SMX-SXT-ERY	4	3	9	0.21
AMP-CZO-SMX-ERY		4	23	
OFL-SMX-SXT-ERY		3	1	
AMP-TET-SMX-SXT		3	1	
AMP-TET-DOX-SMX		3	1	
AMP-FEP-SMX-SXT		3	1	
AMP-FEP-SMX-ERY		4	5	
AMP-CZO-FEP-SMX		3	1	
AMP-CZO-SMX-SXT		3	1	
CZO-SMX-SXT-ERY		3	1	
AMP-TET-SMX-SXT-ERY	5	4	2	0.26
AMP-CZO-FEP-SMX-SXT		3	2	
AMP-SMX-SXT-AZM-KAN		4	1	
AMP-SMX-SXT-ERY-KAN		4	1	
AMP-CZO-SMX-SXT-ERY		4	10	
AMP-CZO-TET-SMX-ERY		5	1	
AMP-CZO-FOX-CTX-SMX		3	1	
AMP-CZO-FEP-SMX-ERY		4	2	
AMP-FEP-SMX-SXT-ERY		4	1	
AMP-CZO-CTX-FEP-SMX-ERY	6	4	1	0.32
AMP-CZO-FEP-SMX-SXT-ERY		4	1	
AMP-IPM-CZO-SMX-ERY-AZM		5	1	
AMP-CZO-TET-SMX-SXT-ERY		5	1	
AMP-CZO-FOX-CTX-FEP-TET-SMX-SXT	8	4	1	0.42
AMP-CZO-FOX-CTX-FEP-TET-SMX-SXT-ERY	9	5	1	0.47

## Data Availability

The original contributions presented in this study are included in the article. Further inquiries can be directed to the corresponding author.

## Appendix A

**Table A1:** Statistical classification table of samples in different quarters and different districts.

Type of Samples (Total Sample Numbers)											
Sample Numbers in Different Quarters				Sample Numbers in Different Districts							
Spring	Summer	Autumn	Winter	Jinshan District	Fengxian District	Qingpu District	Chongming District	Pudong New District	Jiading District	Baoshan District	Songjiang District
Greenhouse-cultured shrimp (*n* = 13)											
4	6	3	-	7	6	-	-	-	-	-	-
Greenhouse-cultured shrimp culture water (*n* = 13)											
5	5	3	-	6	7	-	-	-	-	-	-
Greenhouse-shrimp culture sediment (*n* = 13)											
5	5	3	-	6	7	-	-	-	-	-	-
Open-air-cultured shrimp (*n* = 24)											
4	14	6	-	13	11	-	-	-	-	-	-
Open-air-cultured shrimp culture water (*n* = 25)											
4	14	7	-	15	10	-	-	-	-	-	-
Open-air-cultured shrimp culture sediment (*n* = 25)											
4	14	7	-	15	10	-	-	-	-	-	-
Grass carp (*n* = 18)											
3	5	5	5	2	1	14	-	-	1	-	-
Crucian carp (*n* = 17)											
3	3	5	6	3	1	12	-	-	1	-	-
Fish culture water (*n* = 27)											
6	5	5	11	5	2	18	-	-	2	-	-
Fish culture sediment (*n* = 25)											
4	5	5	11	5	-	18	-	-	2	-	-
Crab (*n* = 10)											
-	-	10	-	-	-	3	3	2	-	1	1
Crab culture water (*n* = 14)											
-	-	14	-	-	-	3	5	3	-	2	1
Crab culture sediment (*n* = 14)											
-	-	14	-	-	-	3	5	3	-	2	1
Total (*n* = 238)											

**Table A2:** Primers of *V. cholerae* for virulence genes, serotype genes, and antimicrobial resistance genes.

Primer Name	Sequences (5′–3′), F/R	Annealing Temperature (°C)	Fragment Size (bp)	References
Serotype				
O1	GTTTCACTGAACAGATGGG	59	192	[51]
	GGTCATCTGTAAGTACAAC			
O139	AGCCTCTTTATTACGGGTGG	59	449	[51]
	GTCAAACCCGATCGTAAAGG			
Virulence gene				
*ctxA*	CTCAGACGGGATTTGTTAGGCACG	55	302	[8]
	TCTATCTCTGTAGCCCCTATTACG			
*ctxB*	GGTTGCTTCTCATCATCGAACCAC	58	460	[8]
	GATACACATAATAGAATTAAGGAT			
*ctxAB*	TGAAATAAAGCAGTCAGGTG	50	778	[19]
	GGTATTCTGCACACAAATCAG			
*tcpA*	ATGCAATTATTAAAACAGCTTTTTAAG	52	675	[19]
	TTAGCTGTTACCAAATGCAACAG			
*ace*	GCTTATGATGGACACCCTTTA	55	283	[8]
	GTTTAACGCTCGCAGGGCAAA			
*zot*	CACTGTTGGTGATGAGCGTTATCG	56	244	[8]
	TTTCACTTCTACCCACAGCGCTTG			
*hap*	GTGAACAACACGCTGGAGAA	50	700	[8]
	CGTTGATATCCACCAAAGG			
*hlyA*	CAATCGTTGCGCAATCGCG	50	265	[8]
	TTGACCTTCAGCATCACT			
*rtxA*	GATTCTTCCGTTCAAGCTCCG	60	2751	[8]
	TGGTTCAGGCTGTTGCACAC			
*rtxC*	CGACGAAGATCATTGACGAC	50	263	[8]
	CATCGTCGTTATGTGGTTGC			
*ompU*	CCAAAGCGGTGACAAAGC	50	655	[8]
	TTCCATGCGGTAAGAAGC			
*chxA*	TGGTGAAGATTCTCCTGCAA	50	421	[52]
	CTTGGAGAAATGGATGCGCTG			
*mshA*	AAAAGTCGACAGCGAAAGCGAATAGTGG	50	380	[52]
	AAAAGGATCCATTGCACCAGCAACTGCACC			
*pilA*	GCGATTGCAATTCCTCAA	56	227	[19]
	CCTAATGCACCTGATGCT			
*stn/sto*	TCGCATTTAGCCAAACAGTAGAAA	50	172	[52]
	GCTGGATTGCAACATATTTCGC			
Antimicrobial resistance gene				
β-lactams				
*CARB*	CAAGTACTTTYAAAACAATAGC	48	535	[53]
	GCTGTAATACTCCKAGCAC			
*bla* *SHV*	GCGAAAGCCAGCTGTCGGGC	62	304	[54]
	GATTGGCGGCGCTGTTATCGC			
*bla* *TEM*	ATAAAATTCTTGAAGACGAAA	50	1080	[55]
	GACAGTTACCAATGCTTAATC			
*NDM-1*	GGTTTGGCGATCTGGTTTTC	52	621	[56]
	CGGAATGGCTCATCACGATC			
*bla* *CTX-M*	CGCTTTGCGATGTGCAG	52	550	[55]
	ACCGCGATATCGTTGGT			
*AmpC*	TGATTAGCGTGTCCTATGGTGA	60	1373	[57]
	GTCAGTAAGGCCCCCTGTTT			
Sulfonamides				
*sul1*	AGGGGGC AGATGTGATCGC	55	626	[58]
	TGTGCGGATGAAGTCAGCTCC			
*sul2*	TCCGGTGGAGGCCGGTATCTGG	55	191	[58]
	CGGGAATGCCATCTGCCTTGAG			
*sul3*	TCCGTTCAGCGAATTGGTGCAG	58	128	[59]
	TTCGTTCACGCCTTACACCAGC			
*sulA*	TCTTGAGCAAGCACTCCAGCAG	58	299	[59]
	TCCAGCCTTAGCAACCACATGG			
*dfrA1*	CAAGTTTACATCTGACAATGAGAACGTAT	60	277	[60]
	ACCCTTTTGCCAGATTTGGTA			
*dfrA18*	ACTGCCGTTTTCGATAATGTGG	60	389	[60]
	TGGGTAAGACACTCGTCATGGG			
Macrolides				
*ermB*	AAAACTTACCCGCCATACCA	55	141	[54]
	TTTGGCGTGTTTCATTGCTT			
*ermC*	GAAATCGGCTCAGGAAAAGG	55	185	[59]
	TAGCAAACCCGTATTCCACG			
*mefA*	ATACCCCAGCACTCAATTCG	56	186	[61]
	CAATCACAGCACCCAATACG			
*mefC*	GCTCCGGCACTACAGGCAAT	56	117	[62]
	GCCAATGGCAGGACCACCAA			
Aminoglycoside				
*aph3a*	TAACAGCGATCGCGTATTTCG	52	250	[62]
	TCCGACTCGTCCAACATCAATA			
*aac(6′)-Ib*	TATGAGTGGCTAAATCGAT	55	395	[63]
	CCCGCTTTCTCGTAGCA			
*armA*	AGGTTGTTTCCATTTCTGAG	53	591	[64]
	TCTCTTCCATTCCCTTCTCC			
*rmtB*	ATGAACATCAACGATGCCCT	56	769	[63]
	CCTTCTGATTGGCTTATCCA			
*aadA*	ATCCTTCGGCGCGATTTTG	56	283	[65]
	GCAGCGCAATGACATTCTTG			
*aadE*	ATGGAATTATTCCCACCTGA	50	386	[65]
	TCAAAACCCCTATTAAAGCC			
Tetracyclines				
*tetA*	GCGCTNTATGCGTTGATGCA	62	387	[59]
	ACAGCCCGTCAGGAAATT			
*tetB*	TACGTGAATTTATTGCTTCGG	60	206	[66]
	ATACAGCATCCAAAGCGCAC			
*tetM*	TCAGTGGGAAAATACGAAGGTG	56	140	[59]
	GAGTTTGTGCTTGTACGCCATC			
*tetO*	ACGGARAGTTTATTGTATACC	56	171	[59]
	TGGCGTATCTATAATGTTGAC			
*tetQ*	AGAATCTGCTGTTTGCCAGTG	56	169	[59]
	CGGAGTGTCAATGATATTGCA			
*tetS*	GAAAGCTTACTATACAGTAGC	56	170	[59]
	AGGAGTATCTACAATATTTAC			
*tetW*	GAGAGCCTGCTATATGCCAGC	56	168	[59]
	GGGCGTATCCACAATGTTAAC			
*tetX*	AGCCTTACCAATGGGTGTAAA	56	280	[59]
	TTCTTACCTTGGACATCCCG			
Chloramphenicol				
*floR*	CGCCGTCATTCCTCACCTTC	50	215	[67]
	GATCACGGGCCACGCTGTGTC			
*cmlA*	GCCAGCAGTGCCGTTTAT	55	158	[68]
	GGCCACCTCCCAGTAGAA			
*catI*	GGTGATATGGGATAGTGTT	52	348	[53]
	CCATCACATACTGCATGATG			
*catII*	GATTGACCTGAATACCTGGAA	54	567	[53]
	CCATCACATACTGCATGATG			
*catIII*	CCATACTCATCCGATATTGA	52	275	[53]
	CCATCACATACTGCATGATG			
*catIV*	CCGGTAAAGCGAAATTGTAT	54	451	[53]
	CCATCACATACTGCATGATG			

**Table A3:** Experimental antimicrobial selection and antimicrobial break point.

Classes of Antimicrobials		Antimicrobials	Concentration of Solution (μg/mL)	MIC of *Vc* (μg/mL)		
				Susceptible	Intermediate	Resistant
β-lactams	Penicillins	Ampicillin (AMP)	5120	≤8	16	≥32
	Carbapenems	Meropenem (MEM)	5120	≤1	2	≥4
		Imipenem (IPM)	10240	≤1	2	≥4
	1st-Generation Cephalosporins	Cefazolin (CZO)	5120	≤2	4	≥8
	2nd-Generation Cephalosporins	Cefoxitin (FOX)	5120	≤8	16	≥32
	3rd-Generation Cephalosporins	Cefotaxime (CTX)	5120	≤1	2	≥4
	4th-Generation Cephalosporins	Cefepime (FEP)	5120	≤2	4-8	≥16
Tetracyclines		Tetracycline (TET)	5120	≤4	8	≥16
		Doxycycline (DOX)	5120	≤4	8	≥16
Chloramphenicol		Chloromycetin (CHL)	5120	≤8	16	≥32
Sulfonamides		Sulfamethoxazole (SMX)	4864	≤38	-	≥76
		Cotrimoxazole (SXT)	4864/256	38/2	-	76/4
Macrolides		Erythromycin (ERY)	5120	≤0.5	1-4	≥8
		Azithromycin (AZM)	5120	≤2	4	≥8
Aminoglycoside		Gentamycin (GEN)	5120	≤4	8	≥16
		Kanamycin (KAN)	5120	≤16	32	≥64
Nitrofurans		Nitrofurantoin (NIT)	5120	≤32	64	≥128
